# Disseminated nontuberculous mycobacteriosis and fungemia after second delivery in a patient with MonoMAC syndrome/GATA2 mutation: a case report

**DOI:** 10.1186/s12879-021-06203-7

**Published:** 2021-05-29

**Authors:** Mizuki Haraguchi, Norihiro Harada, Junko Watanabe, Hitomi Yoshikawa, Yukina Shirai, Moegi Komura, Mika Koyama, Jun Ito, Yutaka Tsukune, Yoshiya Horimoto, Takuo Hayashi, Tetsutaro Nagaoka, Toshimasa Uekusa, Kazuhisa Takahashi

**Affiliations:** 1 Department of Respiratory Medicine, Juntendo University Faculty of Medicine and Graduate School of Medicine, 3-1-3 Hongo, Bunkyo-ku, Tokyo, 113-8431 Japan; 2 Clinical Research Center for Allergy and Rheumatology, National Hospital Organization, Sagamihara National Hospital, Kanagawa, Japan; 3 Research Institute for Diseases of Old Ages, Juntendo University Faculty of Medicine and Graduate School of Medicine, Tokyo, Japan; 4 Department of Hematology, Juntendo University Faculty of Medicine and Graduate School of Medicine, Tokyo, Japan; 5 Department of Human Pathology, Juntendo University Faculty of Medicine and Graduate School of Medicine, Tokyo, Japan; 6 Department of Pathology, Labour Health and Welfare Organization Kanto Rosai Hospital, Kanagawa, Japan

**Keywords:** Fungemia, *GATA2* mutation, MonoMAC syndrome, Nontuberculous mycobacteriosis

## Abstract

**Background:**

Heterozygous mutations in the transcription factor GATA2 result in a wide spectrum of clinical phenotypes, including monocytopenia and *Mycobacterium avium* complex (MAC) infection (MonoMAC) syndrome. Patients with MonoMAC syndrome typically are infected by disseminated nontuberculous mycobacteria, fungi, and human papillomavirus, exhibit pulmonary alveolar proteinosis during late adolescence or early adulthood, and manifest with decreased content of dendritic cells (DCs), monocytes, and B and natural killer (NK) cells.

**Case presentation:**

A 39-year-old woman was diagnosed with MonoMAC syndrome postmortem. Although she was followed up based on the symptoms associated with leukocytopenia that was disguised as sarcoidosis with bone marrow involvement, she developed disseminated nontuberculous mycobacterial infection, fungemia, and MonoMAC syndrome after childbirth. Genetic testing revealed a heterozygous missense mutation in GATA2 (c.1114G > A, p.A372T). Immunohistochemistry and flow cytometry showed the disappearance of DCs and decreased frequency of NK cells in the bone marrow, respectively, after childbirth.

**Conclusions:**

To the best of our knowledge, this is the first study reporting that MonoMAC syndrome can be exacerbated after childbirth, and that immunohistochemistry of bone marrow sections to detect decreased DC content is useful to suspect MonoMAC syndrome.

## Background

Inherited or sporadic heterozygous mutations in the transcription factor GATA2 (guanine-adenine-thymine-adenine 2) causes a germline disease manifesting a wide spectrum of clinical phenotypes including monocytopenia and *Mycobacterium avium* complex (MAC) infection (MonoMAC) syndrome [1, 2]; dendritic cell (DC), monocyte, B and natural killer (NK) lymphoid (DCML) deficiency [3]; familial myelodysplastic syndrome (MDS)/acute myeloid leukemia (AML) [4]; Emberger syndrome (congenital deafness, primary lymphedema, and predisposition to MDS and AML) [5]; and classic NK cell deficiency [6]. Patients typically develop MonoMAC syndrome and DCML deficiency during late adolescence or early adulthood as a result of opportunistic infections, such as disseminated nontuberculous mycobacterial (NTM) infections, fungal infections, and severe or recurrent human papillomavirus infections, pulmonary alveolar proteinosis, and decrease in DCs, monocytes, and NK and B cells [1, 7, 8]. These patients are associated with an increased risk of MDS and AML [2].

Herein, we report a female patient with leukocytopenia which was disguised as a sarcoidosis with bone marrow involvement. She was followed up for 7 years to assess the accuracy of the diagnosis and developed disseminated NTM infection and fungemia along with MonoMAC syndrome after childbirth. To the best of our knowledge, this is the first report on the importance of immunohistochemistry of bone marrow to detect decreased DC content to suspect MonoMAC syndrome.

## Case presentation

A 39-year-old Japanese female presented with high fever 1 week after her second childbirth by Cesarean section. She subsequently developed acute pericarditis with dyspnea, bilateral pleural effusion, pericardial effusion, ascites, systemic lymphadenopathy, hepatosplenomegaly, and leukopenia. Acute pericarditis was improved spontaneously after admission to our cardiology department. However, her fever continued and leukopenia worsened. Complete blood counts showed pancytopenia with 800 leukocytes/μL (32 monocytes/μL), hemoglobin content of 9.9 g/dL, and 108,000 platelets/μL 50 days postpartum (Table 1). Bone marrow aspirates and biopsy showed normal cellularity and non-caseating granuloma, without myelodysplastic features.

**Table 1:** Results of blood examinations and bone marrow aspirations

		Postpartum period						
**Bone marrow aspiration**	**Reference range**	**−6.3 years**	**− 6 years**			**56 days**	**5 months**	
T cells (CD3+), % of lymphocytes	59–88	45.3	95.7			56.6	73.7	
CD4 + T cells, % of lymphocytes	29–65	36.1	n/a			n/a	15	
CD8 + T cells, % of lymphocytes	13–40	26.7	n/a			n/a	49.6	
CD4/CD8 ratio	0.6–2.9	1.4	n/a			n/a	0.3	
B cells (CD19+), % of lymphocytes	4–26	1.1	1.3			1.2	1.4	
NK cells (CD3-CD56+), % of lymphocytes	2–26	9.3	n/a			0.3	0.3	
**Complete blood counts**	**Reference range**	**−6.3 years**	**−6 years**	**−3 years**	**19 days**	**50 days**	**5 months**	**9 months**
White blood cells, cells/μL	3600–8900	1800	2000	1900	1400	800	700	200
Neutrophils, %	37–72	30.5	32.5	39	43	42	79	n/a
Lymphocytes, %	25–48	53.5	55.5	44	51	52	12	n/a
Monocytes, %	2–12	14	12	16	4	4	3	n/a
Monocytes, cells/μL		252	240	304	56	32	21	n/a
Hemoglobin, g/dL	11.1–15.2	12.1	11.8	9.6	10	9.9	9.7	5.7
Platelets, 10^3^ counts/μL	153–346	92	88	92	176	108	74	10
**Serum**	**Reference range**		**−6 years**		**19 days**	**83 days**	**5 months**	**9 months**
IgG, mg/dL	870–1700		1304		1081	858	540	57

*Abbreviations: n/a* not applicable, *NK* natural killer

**Fig. 1:**
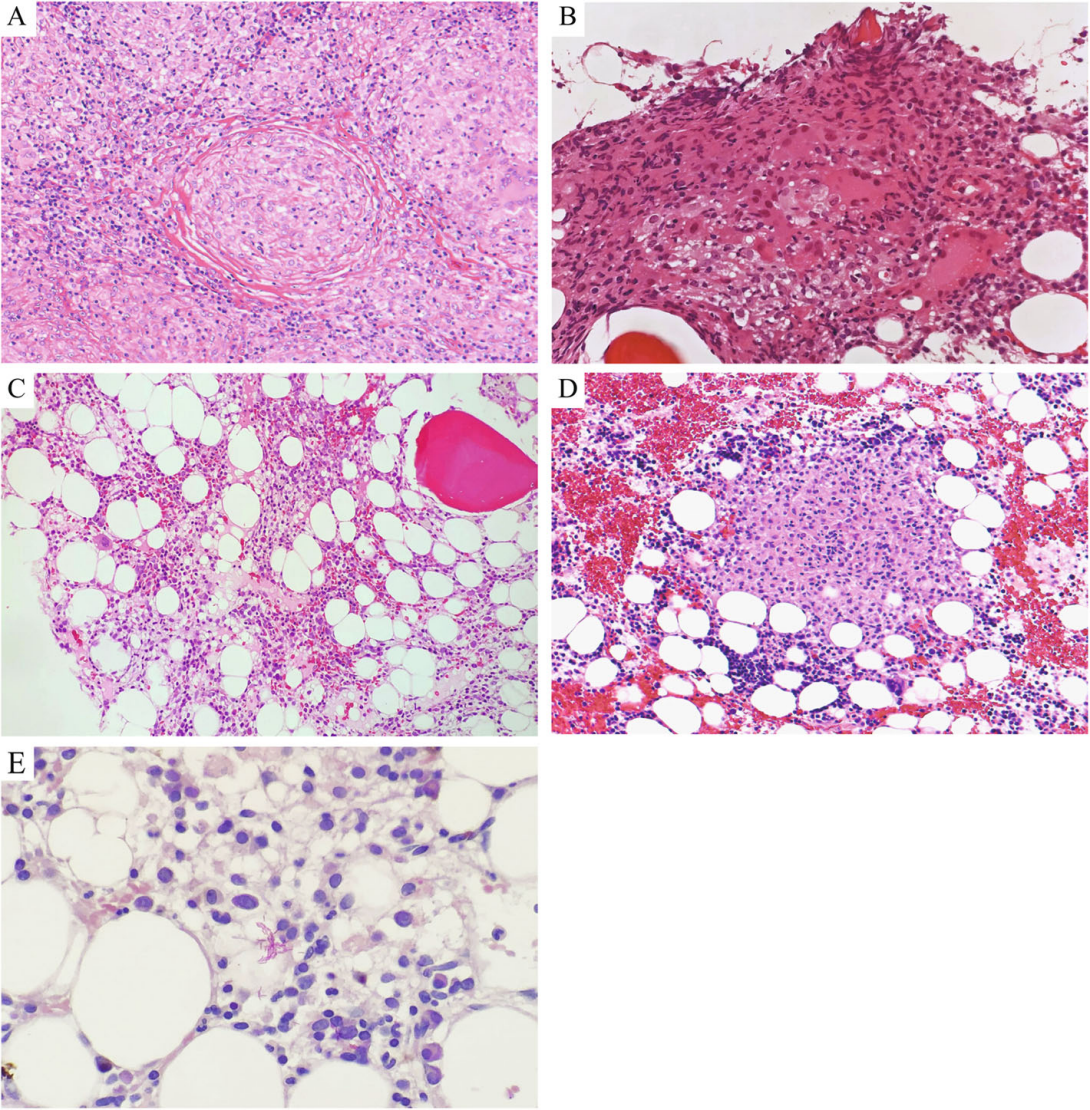
Histopathological analysis of the cervical lymph node biopsy (**a**) and bone marrow biopsy specimens (**b** and **c**) 7.5 and 6.5 years before giving birth, respectively and 56 days postpartum (**d** and **e**). Normal cellularity and non-caseating granulomas were observed (hematoxylin and eosin staining, magnification × 200; **a**, **c**, and **d**, × 400; **b**). Ziehl-Neelsen-positive stained sections (magnification × 400; **e**)

The patient was the second child born to unrelated parents. She was healthy until 31 years of age except for an ectopic pregnancy at 28 years and recurrent warts; during this time, she gradually developed lymphadenopathy on her neck. Moreover, her peripheral blood showed mild pancytopenia. She was histopathologically diagnosed, at 32 years, with sarcoidosis with involvement of the cervical lymph node (Fig. 1a) and bone marrow (Fig. 1b), based on the presence of non-caseating granulomas with a background of normocellular bone marrow (Fig. 1c). Ziehl–Neelsen staining was negative for both the lymph node and bone marrow specimens (data not shown). Cytogenetic analysis of the bone marrow cells showed a normal 46 XX chromosomal pattern. The results of the bone marrow aspiration and peripheral blood counts obtained 6.3 years before the second childbirth of the patient are shown in Table 1. Although cytopenia was observed, there were neither symptoms nor signs of deterioration in the laboratory findings, and she was suspended from hospital visits. She presented again with regrowth of cervical lymphadenopathy during pregnancy at 36 years, her peripheral blood counts at the time are shown in Table 1 as “3 years before her second childbirth” (Table 1). After her first childbirth, the lymphadenopathy disappeared spontaneously. Moreover, her bone marrow sarcoidosis was believed to have caused leukopenia. She was followed up without treatment (Table 1).

**Fig. 2:**
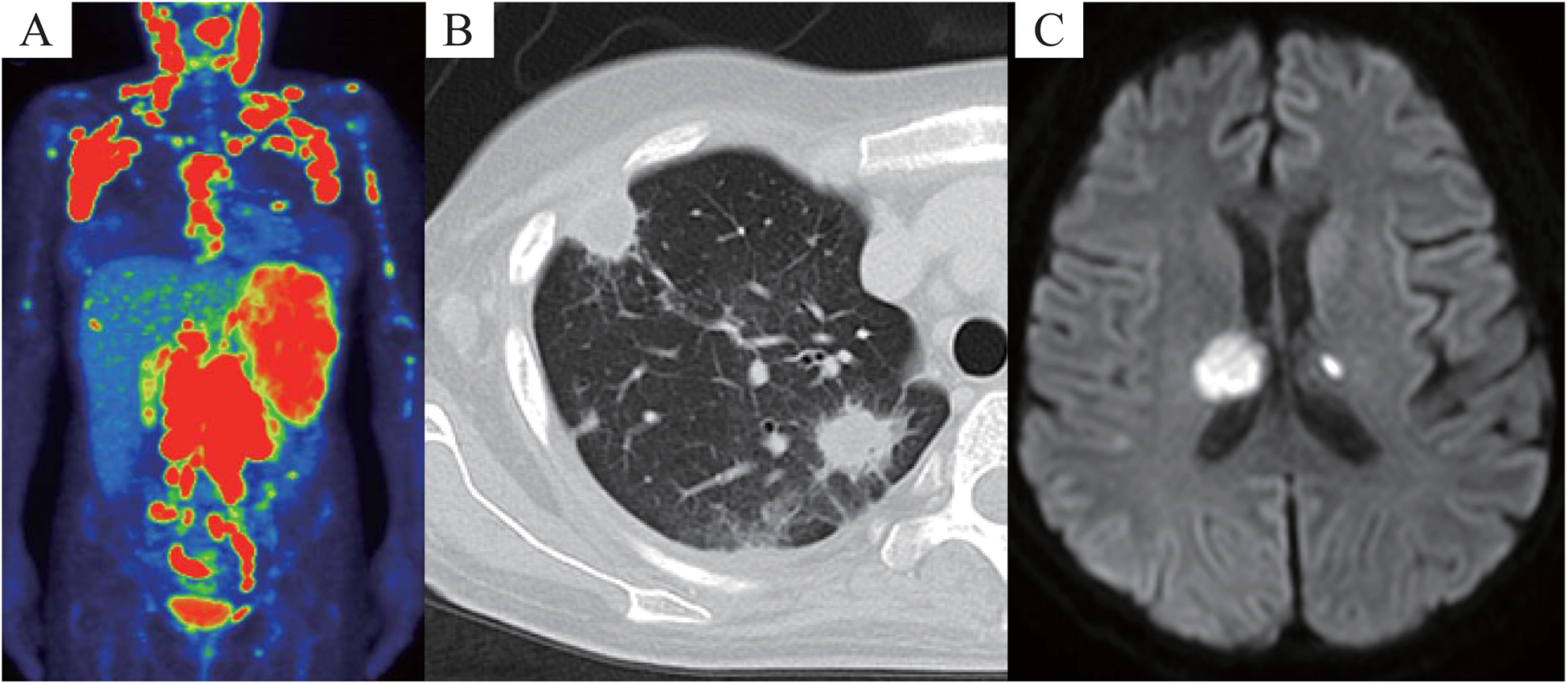
Scans obtained from ^18^F-fluorodeoxyglucose positron emission tomography-computed tomography (**a**), chest computed tomography (**b**), and head magnetic resonance imaging (**c**). An increase in the uptake of fluorodeoxyglucose in the cervical, axillary, mediastinal, and mesenteric lymph nodes, spleen, and bone marrow was observed 42 days postpartum (**a**). Multiple nodular shadows and pleural effusion were observed in both fields 9 months postpartum (**b**). Diffusion weighted image axial brain magnetic resonance imaging showing the evolution of cerebral infarction 9 months postpartum (**c**)

Monitoring the whole-body uptake of ^18^F-fluorodeoxyglucose using positron emission tomography-computed tomography revealed hepatosplenomegaly and increased diffuse uptake of fluorodeoxyglucose in the systemic lymph nodes, spleen, and bone marrow (Fig. 2a). We suspected sarcoidosis and malignant lymphoma owing to the high levels of angiotensin converting enzyme (84 U/L), lactate dehydrogenase (1436 IU/L), and soluble interleukin-2 receptor (10,100 U/mL), positron emission tomography-computed tomography scans, and previous pathological findings. Since she suffered from prolonged fever and severe general exhaustion, an axillary lymph node biopsy was performed to confirm the diagnosis, and steroid therapy (prednisone, 1 mg/kg) for sarcoidosis was initiated 86 days postpartum. Her clinical condition, fever, and general condition improved after 2 weeks of steroid therapy along with reduction in hepatosplenomegaly. Lymph node biopsy showed reactive histiocytic hyperplasia that did not enable a definitive diagnosis. Fever, splenomegaly, and leukopenia reappeared while tapering the steroid therapy. We performed bone marrow biopsy again to detect normal cellularity and non-caseating granulomas with positive Ziehl-Neelsen staining (Fig. 1d and e). Ziehl-Neelsen staining also detected a small population of mycobacteria in the pathological specimens from an axillary lymph node during retrospective analysis, and blood culture revealed the presence of *M. avium*. Disseminated NTM infection was diagnosed and combination therapy with clarithromycin, rifampicin, and ethambutol was administered 6 months postpartum. However, owing to liver dysfunction and acute kidney injury, the treatment regimen was modified to ethambutol, kanamycin, azithromycin, and levofloxacin. She developed fungemia (*Candida glabrata*) along with sudden impaired consciousness, and a magnetic resonance imaging of the head revealed multiple cerebral infarctions, suggesting septic emboli. Unfortunately, despite administering the patient with the specific therapeutics for these diseases, she manifested with progressing disseminated fungal and bacterial infection (Fig. 2b and c). Ultimately, the patient exhibited respiratory failure and died 9 months postpartum.

**Fig. 3:**
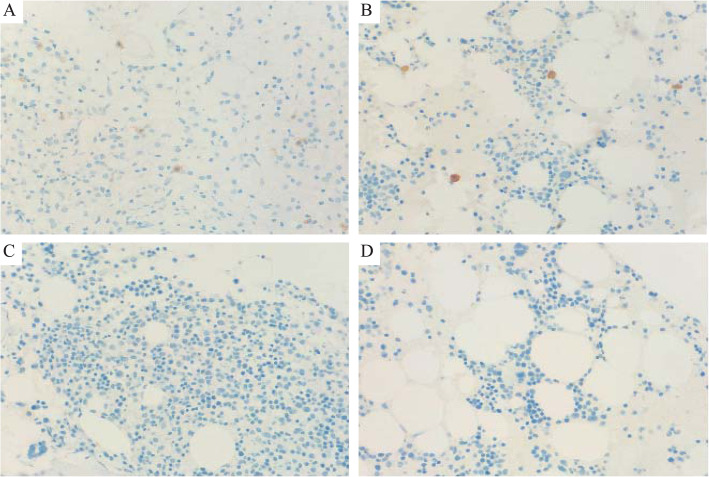
Postmortem immunohistochemical analysis of bone marrow biopsy specimens 6.5 years before giving birth (**a**), 6 years before giving birth (**b**), 56 days postpartum (**c**), and 5 months postpartum (**d**). These specimens were positive for CD21 (**a** and **b**) and negative for CD21 (magnification × 400; **c** and **d**)

Anatomical pathology and fs revealed systemic invasion of the brain, heart, lung, liver, stomach, intestine, spleen, lymph nodes, and bone marrow by *Aspergillus flavus*, *A. fumigatus*, *Candida glabrata*, and *M. avium*. The cause for immunodeficiency was analyzed while the patient was being treated for disseminated NTM infection. Genetic testing showed the presence of a heterozygous missense mutation in *GATA2* (c.1114G > A, p.A372T) confirming GATA2 deficiency. Furthermore, postmortem immunohistochemical analysis showed that DCs disappeared and the NK cell population decreased in the bone marrow after childbirth (Table 1 and Fig. 3).

## Discussion and conclusions

Here, we present, to the best of our knowledge, the first case of a patient with MonoMAC syndrome that may have exacerbated after childbirth, and we showed that immunohistochemistry of bone marrow sections to detect the decrease in DC population can lead to suspicion of MonoMAC syndrome. The patient developed disseminated MAC and fungal infection (*A. flavus, A. fumigatus, and C. glabrata*), and died of multiple acute cerebral infarctions. After death, we detected heterozygous missense mutations in *GATA2* and confirmed the diagnosis of MonoMAC syndrome. There are three known cases of the same mutation (c.1114G > A,p.A372T in *GATA2*) [7, 9, 10]. The patient’s parents and older brother did not possess an increased susceptibility to infection and her brother tested negative for mutations in *GATA2*. Although her mother was diagnosed with MDS and bearing trisomy 8 at 51 years, the patient did not have any recurrent infections, nor shared the exact genetic information of the chromosomal aberration among her family. Hence, the study of her chromosomal abnormalities was difficult owing to these factors. After a postmortem genetic test revealed her GATA2 deficiency, her mother was also tested and was found to have the same mutation in GATA2. Her mother did not exhibit immunodeficiency and was followed up by our hematology department. Thus far, four large surveys have reported a total of 232 patients with germline mutations in *GATA2* [9–12]. Mycobacterial and fungal infection can be found in 42–53% and 16–20% of patients with GATA2 deficiency, respectively [10, 11]. Donadieu et al. reported that 34% of patients die at a median age of 29 years (range, 10.2–72.6 years) [10]. A poor survival rate was observed after the disease onset (first severe infection, myelodysplasia/leukemia, pulmonary alveolar proteinosis, or lymphedema), and the rates of mortality are 4–6%, 23–42%, and 55–69% at 20, 40, and 60 years of age [10, 11]. Patients with GATA2 deficiency acquire chromosomal abnormalities, such as monosomy 7 and trisomy 8, during disease progression of myelodysplasia. Furthermore, 48–65% of these patients manifest with abnormal bone marrow cytogenetics, including trisomy 8 in 9–24% of the individuals [10–13].

During healthy pregnancy, the immune system needs to maintain tolerance to the fetal allograft. This involves the suppression of CD4^+^ T helper type (Th) 1 activity and upregulation of Th2 cells and regulatory T cells (Tregs) [14]. Th1/Th17-dominant autoimmune diseases tend to ameliorate during pregnancy but flare up postpartum, whereas Th2-dominant diseases, including asthma, tend to be exacerbated by pregnancy or childbirth [15, 16]. Postpartum exacerbation of Th1/Th17-dominant diseases is caused by the decrease in Th2 and Tregs [14]. These dramatic changes in the T cell immune balance during pregnancy may have prevented the return to the pre-pregnancy state after childbirth due to GATA2 dysfunction. As a result, DCs and Tregs would decrease, reducing the resistance of the T-cell immunity to infection and generating excessive inflammation, respectively. GATA2 dysfunction may impair T cell differentiation from hematopoietic stem cells, resulting in a decrease in the number of T cells supplied to peripheral blood. Th17 cells are important for defense against fungal infections, and it is known that impaired differentiation of IL-17-producing T cells is related with a compromised immunity against fungal infections [17]. Treg decrease may be associated with a decrease in DC in GATA2 deficiency [18, 19]. Approximately 11% of patients with GATA2 deficiency manifest autoimmune diseases, such as lupus and sarcoidosis-like disease [10]. Moreover, hormone-controlled T cell responses influence the development of autoimmune diseases: progesterone and estrogens induce Th2 response and decrease Th1/Th17 response [14, 20–22], and estrogen enhances B cell antibody production, reducing B and T lymphopoiesis and inhibiting T cell-dependent inflammation [14, 23]. Although MonoMAC syndrome may have exacerbated in our patient after childbirth, there is no reported case of MonoMAC syndrome and immunodeficiency exacerbated by pregnancy or childbirth. Spinner et al. reported that 14 (33%) of 43 pregnancies with GATA2 deficiency result in miscarriage, while 15–20% of the general population experience miscarriage [11]. Several cases of sarcoidosis-like granulomatous disease have been reported in patients with *GATA2* mutations (aside from A732T) [9–11, 24]. Similar to our patient, a 42-year-old individual with mutant *GATA2* was diagnosed with histiocytosis X and died of MAC infection [24].

Mutations in *GATA2* lead to a wide spectrum of immunological phenotypes with decrease or deficiency of monocytes, DCs, B cells, and NK cells [1–3, 6, 7, 25]. Donadieu et al. have used immunological data from 38 patients to report: slight decreases in T-cell counts; preservation of NK cells; and low B-cell levels, although immunoglobulin levels were within normal ranges [10]. In this case, postmortem retrospective analysis showed deficiency of B cells and the decrease in DCs, NK cells, and CD4/8 ratio of T cells in the bone marrow and reduction of monocytes in peripheral blood after childbirth (Table 1 and Fig. 3). As in previous reports, her immunoglobulin levels were within the normal ranges until the infections became severe [10]. GATA2 deficiency was suspected owing to reduced B-cell levels, warts, granulomas, and histiocytic hyperplasia. Decreased content of monocytes, NK cells, DCs, and CD4/8 ratio in the peripheral blood and bone marrow were considered to be associated with postnatal deterioration (Table 1 and Fig. 3). Although DCs are usually analyzed by flow cytometry [18], we were only able to confirm their phenotype using pathological analysis (Fig. 3). Conversely, monocytes were evaluated using peripheral blood and not bone marrow (Table 1). Therefore, the lack of sufficient data on DCs and monocytes serves as a limitation for this report.

There are some other limitations of this case report. Although the post-mortem diagnosis provided certain information that led to a retrospective diagnosis, we could not use this information to make an accurate diagnosis during the lifetime of the patient. Another limitation is the lack of data of the 6 years after diagnosis of sarcoidosis, since the patient attended a medical institution other than our hospital. Moreover, tissue culture was not performed even though repeated biopsies of bone marrow and lymph nodes were performed; to only rely on Ziehl-Neelsen staining was also a limitation of the study.

In conclusion, we have described that the progression of exacerbated MonoMAC syndrome may be associated with childbirth and decreased DC, NK cell, and monocyte content. Moreover, we found that immunohistochemistry of the bone marrow to evaluate DC content is a useful approach to suspect MonoMAC syndrome.

## Data Availability

Data sharing is not applicable to this article as no datasets were generated or analyzed during this case report.

## Abbreviations

AML: acute myeloid leukemia
DC: dendritic cell
DCML: dendritic cell, monocyte, B, and natural killer lymphoid
GATA2: guanine-adenine-thymine-adenine 2
MAC: *Mycobacterium avium* complex
MonoMAC: monocytopenia and MAC infection
MDS: myelodysplastic syndrome
NK: natural killer
NTM: nontuberculous mycobacterial
Th: T helper
